# Real-time imaging of acoustic waves in bulk materials with X-ray microscopy

**DOI:** 10.1073/pnas.2307049120

**Published:** 2023-09-19

**Authors:** Theodor S. Holstad, Leora E. Dresselhaus-Marais, Trygve Magnus Ræder, Bernard Kozioziemski, Tim van Driel, Matthew Seaberg, Eric Folsom, Jon H. Eggert, Erik Bergbäck Knudsen, Martin Meedom Nielsen, Hugh Simons, Kristoffer Haldrup, Henning Friis Poulsen

**Affiliations:** ^a^Department of Physics, Technical University of Denmark, Kongens Lyngby 2800, Denmark; ^b^Department of Materials Science & Engineering, Stanford University, Stanford, CA 94305; ^c^SLAC National Accelerator Laboratory, Menlo Park, CA 94025-7015; ^d^Physics Division, Lawrence Livermore National Laboratory, Livermore, CA 94550-9234

**Keywords:** acoustic waves, phonons, ultrafast imaging, X-ray free electron laser, dark-field X-ray microscopy

## Abstract

The dynamics of lattice vibrations govern many material processes, such as acoustic wave propagation, displacive phase transitions, and ballistic thermal transport. The maximum velocity of these processes and their effects is determined by the speed of sound, which therefore defines the temporal resolution (picoseconds) needed to resolve these phenomena on their characteristic length scales (nanometers). Here, we present an X-ray microscope capable of imaging acoustic waves with subpicosecond resolution within mm-sized crystals. We directly visualize the generation, propagation, branching, and energy dissipation of longitudinal and transverse acoustic waves in diamond, demonstrating how mechanical energy thermalizes from picosecond to microsecond timescales. Bulk characterization techniques capable of resolving this level of structural detail have previously been available on millisecond time scales—orders of magnitude too slow to capture these fundamental phenomena in solid-state physics and geoscience. As such, the reported results provide broad insights into the interaction of acoustic waves with the structure of materials, and the availability of ultrafast time-resolved dark-field X-ray microscopy opens a vista of new opportunities for 3D imaging of materials dynamics on their intrinsic submicrosecond time scales.

## Significance

In this work, we present ultrafast (subpicosecond), submicrometer-resolution direct imaging of phonon propagation in bulk materials. To achieve this, we developed a unique X-ray diffraction microscopy technique for capturing minute lattice perturbations in deeply embedded volumes. This result is significant because phenomena occurring at timescales dictated by lattice dynamics are ubiquitous (e.g., displacive phase transformations, acoustic wave propagation, and ballistic thermal transport), yet the means for nondestructively imaging the structural changes associated with these phenomena have—until now—been six orders of magnitude too slow. The approach is generally applicable to all types of crystalline matter and will therefore be broadly relevant across solid-state physics, as well as materials science and geoscience.

Understanding the structural dynamics of crystalline solids is a key aspect of materials science, geoscience, and solid-state physics. However, the structure of many materials is complex, exhibiting dynamics across multiple length- and time-scales simultaneously. In situ tools for visualization of such multiscale dynamics is in general lacking. As a consequence, materials models and simulations have suffered from a lack of input from experiments and, in many cases, exhibit poor prediction capabilities. In particular, this is the case for structural materials, such as most metals, ceramics, rocks, and bone, where the structure is organized hierarchically in grains, domains, and defects, and competing interactions take place on length scales from nanometers to centimeters (1). For such materials, experimental techniques capable of characterizing samples that are tens or hundreds of micrometers thick are required to build reliable models.

To record movies of the evolution of phase transitions, grain-boundary or domain motion, and crystalline defects/strains within mm-thick specimens, X-ray diffraction–based imaging methods have been developed. Exploiting the brightness and penetration power of synchrotron X-ray sources, modalities include 3D X-ray diffraction (2, 3), diffraction-contrast tomography (4), differential-aperture X-ray structural microscopy (5, 6), and dark-field X-ray microscopy, DFXM (7, 8). With a spatial resolution down to 100 nm, these methods have been used, e.g., to reveal underlying mechanisms in nucleation and growth phenomena (9, 10), in plastic deformation (11), in fracture (12), in phase transformations (13), in dislocation dynamics (14), and in the complex mechanics of bone (15).

Currently, a main limitation of these methods is the time resolution: the limited brightness of the source requires acquisitions over milliseconds to seconds for each single image, making spatially resolved 3D maps require minutes to hours for different sample volumes and imaging modalities. In contrast, many diffusive processes occur on timescales of microseconds, while numerous processes like Martensitic phase transitions, charge-density waves, and thermal transport occur even faster (in pico- to nanoseconds).

In this work, we demonstrate X-ray imaging within the bulk of a material that captures structural dynamics in <100 femtosecond snapshots. Specifically, we visualize the propagation of acoustic waves in a diamond single crystal as it shifts from a mechanical “impulse” into a thermal bath. By applying DFXM at an X-ray free electron laser (XFEL), our movies visualize and quantify the ultrafast propagation of the sound waves and resolve their interactions with surfaces, converting energy into transverse modes that “ring down” the energy over picosecond through microsecond timescales. We include coupled thermomechanical and X-ray optics simulations to illustrate the generation and imaging technique of acoustic waves in this setup (16).

**Fig. 1.**
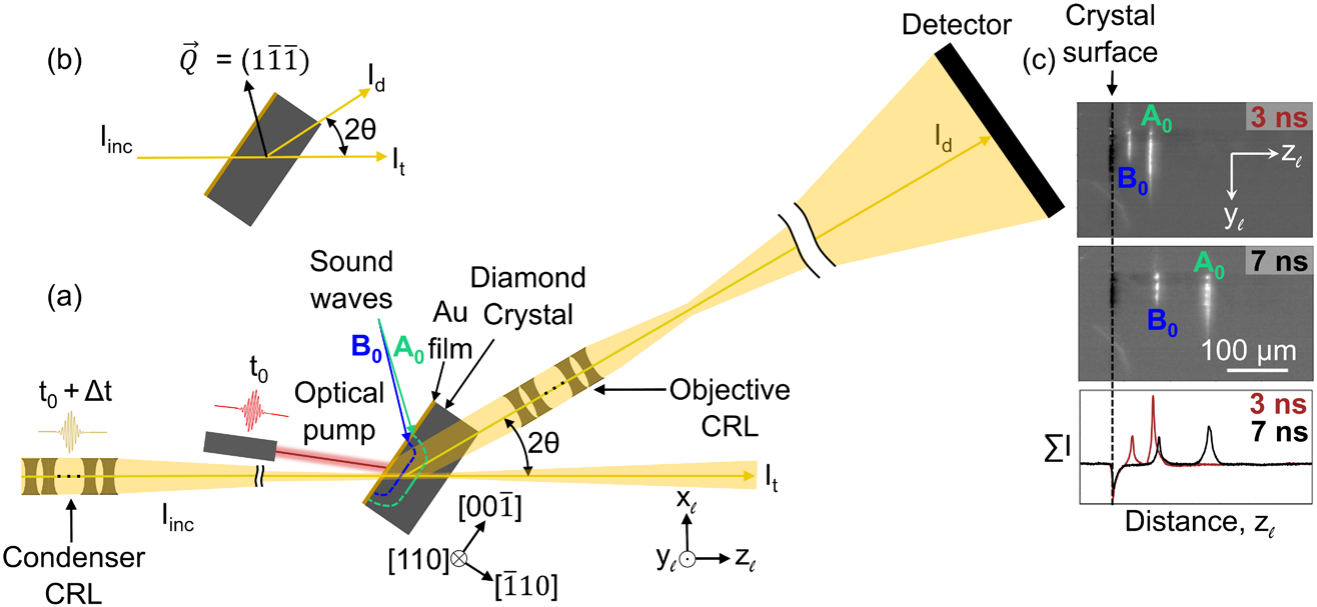
(*A*) Schematic layout of the DFXM experiment. The incident X-ray beam is condensed in one direction using a compound refractive lens (CRL) to illuminate a layer in the sample, which defines the observation plane (horizontal in this figure). The diamond single crystal is oriented such that diffraction takes place on reflection, $\vec{Q}$  . DFXM images of the observation plane are obtained by using an X-ray objective to magnify the Bragg diffracted X-rays onto a 2D detector. An optical laser pump heats a Au film deposited on the surface of the sample at time *t*_0_, leading to thermal expansion and the launching of acoustic waves inside the diamond crystal. The associated local strain variations in the crystalline lattice are imaged at different time delays Δ*t* between the laser pump and X-ray probe to create a movie of their propagation. Facets of the crystal and a laboratory coordinate system are shown. (*B*) Scattering geometry. (*C*) Experimental DFXM images at Δ*t* = 3 ns and 7 ns. Below are graphs of intensity with the vertical *y_ℓ_*-direction integrated out. Two acoustic waves, marked A_0_ (aquamarine) and B_0_ (blue), are seen to propagate toward the right for increasing Δ*t*.

As illustrated in Fig. 1*A*, we use a fs-duration visible laser to pump the acoustic waves, then use the XFEL pulses to probe the dynamics using an objective lens along the X-ray diffracted beam. By illuminating a thin observation plane in the sample, each image provides a magnified view of the subtle distortions in the crystalline lattice within a 2D slice of the macroscopic mm-sized sample. This allows us to resolve how even subtle deviations from phonons and defects generate local variations (strain and orientation changes) in the lattice. In this work, we acquire movies of the associated dynamics by repeating our experiment over a series of time delays, Δ*t*, between the optical pump and X-ray probe pulses.

**Fig. 2.**
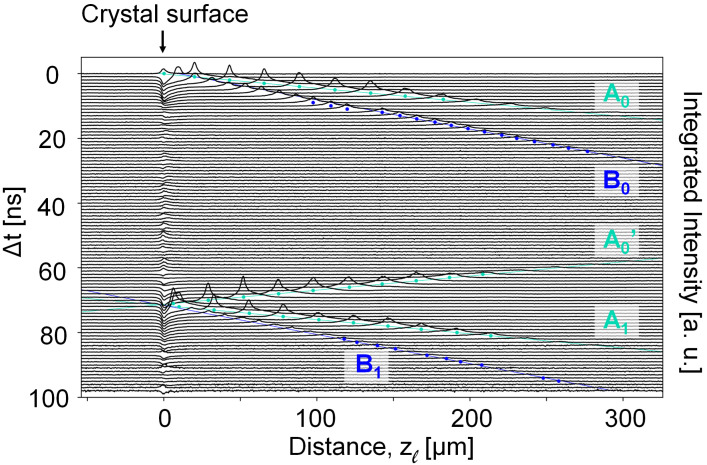
Propagation of the strain waves in diamond over increasing delay times Δ*t*. For each time step, the 1D trace is generated by integrating the intensity over the vertical *y_ℓ_*-direction, cf. Fig. 1*C*. The intensity thus maps the temporally weak-beam signal from the strain waves as function of distance along *z_ℓ_* from the crystal surface, as the wave traverses the observation plane. The plots are vertically offset according to their time delay. The colored dots under each peak signify the positions of each wave’s peak, and the straight aquamarine and blue lines plot the linear fits formed by them.

Snapshots from a movie (Movie S2) spanning time delays of Δ*t* = 0 to 100 ns are shown in Fig. 1*C*. From this movie, it appears that the photoexcitation of the Au film launches two strain waves, labeled *A*_0_ and *B*_0_, respectively. As predicted (*SI Appendix*, Fig. S1), the waves are nearly planar over the region we image. To quantify the position of the waves as a function of time, we integrate the image intensities along the *y_ℓ_*-axis and visualize the amplitude and spatial profile as a function of their position along *z_ℓ_* (Fig. 2). The strain waves A_0_ and B_0_ propagate at different velocities as they travel toward the rear face of the diamond crystal (i.e., to the right in Fig. 2, beyond the image’s field of view). The velocity of the fast wave is 18.21 km/s (*SI Appendix*, section 6), which is consistent with previous reports in the literature of a longitudinal sound velocity of 18.18 ± 0.03 km/s in the *<*110*>* directions in diamond (17).

Assuming the slow wave to propagate in direction [ $\bar{1}$10] as well, the velocity is 8.86 km/s. This is close to a theoretical prediction of 8.95 km/s for the slow transverse acoustic wave along the*<*110*>*-directions (18). The sound velocities determined also correspond well with those obtained by other methods [ultrasound (17), Raman (18), electron diffraction (19)], confirming the ability to quantify dynamical processes.

Strain wave A_0_ reflects off the rear surface of the crystal and returns into the field of view (A_0_^′^). Comparing same positions before and after the component is seen to transmit through the crystal with a nearly soliton structure that scarcely changes the wave’s profile as it propagates. On return to the front Au-coated surface of the crystal, this wave is reflected (A_1_). The multiple “bounces” off each surface of the crystal sets up an acoustic cavity that traps the wave between two reflective surfaces as it dissipates energy into the crystal (20).

**Fig. 3.**
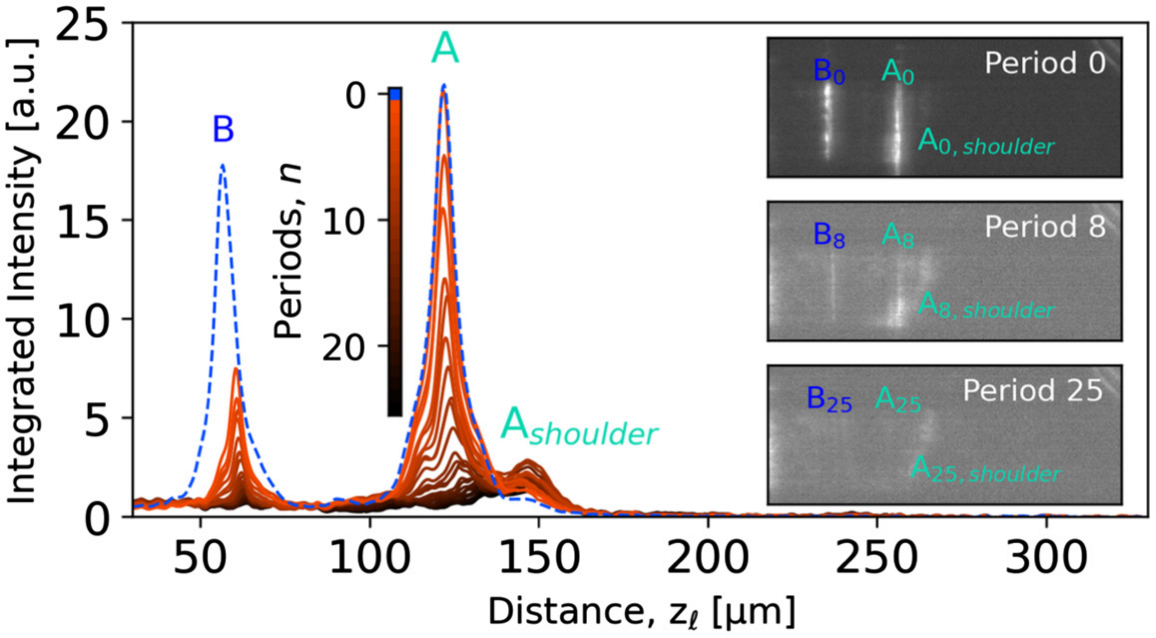
Dispersion of the acoustic waves. Intensity profiles (averaged over *y_ℓ_*) for the fast (A) and slow (B) strains waves at time delays of 5.5 ns + *n*Δ*t_p_*, where Δ*t_p_*= 72.47 ns is the time period for the longitudinal wave to return to the surface from which it was launched. The curves are generated by integration over a vertical region-of-interest, similar to Fig. 2. Representative DFXM images are shown on the right for 0th, 8th, and 25th period.

This is evident from Fig. 3, showing snapshots from a movie (Movie S3) with time delays of Δ*t* = 5.5 ns + *n*Δ*t_p_*, where Δ*t_p_*= 72.47 ns is the “period” corresponding to one round-trip. A total of 26 periods were captured. Fig. 3 also shows that for each time wave A is reflected off the Au-coated surface, a new strain wave B is emitted. The apparent formation of a new mode upon each surface reflection suggests that a fixed fraction of energy is transferred from the longitudinal wave to the new transverse waves, with an associated reflectivity of wave A of 89% per period (*SI Appendix*, Fig. S3). With increasing period number *n*, the intensity profile for strain wave A becomes bimodal. While the intensity of the longitudinal wave A decreases continuously, the relative intensity of the shoulder increases with increasing number of reflections (Fig. 3). We interpret this energy transfer as caused by dispersion taking place during either propagation or reflection, but further experiments are needed to elucidate its exact nature. The contrast in the images in Fig. 1*C* was provided by rotating the sample slightly around the *y_ℓ-_* axis with respect to the Bragg condition of the bulk crystal. By similar rotation around *z_ℓ_* and by varying the scattering angle 2*θ*, three elastic strain components can be mapped by DFXM with a strain resolution better than 10^−4^ (ref. 21).

Determining the direction of displacement of the waves by considering the relation between image-contrast and these rotations corroborates that A is a longitudinal wave (*SI Appendix*, sections 4 and 5). Moreover, we find that the spatial variation of the strain-wave contrast is well described by a combined thermomechanical (22) and X-ray geometrical optics forward simulation (21, 23), see *SI Appendix*, Fig. S8.

This study was performed with an ad hoc experimental setup. With available X-ray optics, the spatial resolution in DFXM at the synchrotron is 100 nm (ref. 8). By translating the sample perpendicularly to the X-ray beam and by varying the sample orientation, movies can be made with a high resolution in both 3D direct space and 3D reciprocal space, enabling, e.g., a full spatiotemporal mapping of the phonon spectrum (see discussion in *SI Appendix*, sections 4 and 5).

Until now, ultrafast work on phonon dynamics and thermalization has primarily been *spectroscopic*, using time-domain Brillouin and Raman scattering (24), or electron and X-ray *diffraction* studies (19, 25–27). However, these studies are not spatially resolved. Spatially resolved studies have thus far been restricted to thin foils using ultrafast electron microscopy (28, 29), to surface acoustic waves (30), or to optically transparent samples (31). In contrast, the method presented above can directly image traveling acoustic waves, generated by packets of phonon modes, in the bulk of optically opaque materials, with a high and symmetry-selective strain sensitivity. It is well suited to probe the interaction between strain waves and structural elements such as dislocations, grain boundaries, or voids, as well as interference between strain waves. This is of critical importance to understand phonon lensing in meta-materials and photonic crystals (32), for thermal engineering studies that seek to control the flow of heat in thermoelectric materials (33), and for understanding phonon behavior in soft materials such, e.g., as perylene (27) and hybrid perovskites (34). We also see opportunities for this approach in geosciences, extending it to polycrystalline materials to test seismological models of sound propagation in planetary materials (35).

Exploiting that acoustic strain wave generation is a reversible process, the results represent averages over several images taken at the same delay time. However, the intensities in the individual raw images before averaging are ∼200 counts/pixel (Movie S1), with a SNR of ∼8.5. Moreover, the X-ray scattering signal from nearly all materials is larger than that of diamond. Hence, each pump event may be followed by not one but a train of hundreds of X-ray probe pulses—as provided by some XFELs. Uniquely, this opens opportunities for visualizing stochastic and *irreversible* structural processes in real-time on the sub-µs timescale. Such processes are ubiquitous in materials science, e.g., martensitic phase transformations in steel, domain switching in ferroelectrics, and dielectric breakdown.

## Methods

The X-ray microscope established at the X-ray Correlation Spectroscopy (XCS) instrument at the LCLS XFEL during beamtime in June 2021 was similar to existing DFXM instruments at synchrotrons (7, 8). The full details of the optical and hardware setup are presented in ref. 36. In brief, the 10.1 keV X-ray pulse was monochromatized to provide an energy bandwidth of Δ*E/E* = 10^−4^. The incident beam was focused horizontally to a thickness of 3.9 µm by a compound refractive lens (CRL) condenser placed 3.43 m before the sample. The objective CRL comprised 33 Be lenslets with a 50-µm radius and a resulting focal length of 0.205 m and a numerical aperture (FWHM) of 8.5 × 10^−4^ (ref. 8). The sample-detector distance was 6.83 m. The 2D detector comprised an Andor Zyla 5.5 sCMOS camera with 6.5-µm pixel size coupled to a scintillator screen by visual optics. The resulting magnification of the diffraction imaging system was determined to be ∼30.

Here, we combine DFXM with a pump–probe scheme. A 300-nm gold film was sputter-coated on the polished ( $\bar{1}$ 10)-facet of an Element 6 diamond single crystal grown via chemical vapor deposition and laser cut to dimensions of 1 × 2 × 0.66 mm^3^ (Fig. 1). A 15-nm Ti film was used as an adhesion layer. An optical laser pump from a Ti:Sapphire laser system with a wavelength of 800 nm, an energy per pulse of 100 µJ, and a pulse duration of 50 fs was used to induce ultrafast heating and expansion within a spot with a diameter of 150 µm (FWHM) in the Au layer, thereby launching strain waves into the diamond. An X-ray probe with an energy per pulse of 1.6 mJ and a pulse duration of 50 fs was used to image the propagation of the strain waves. For the (1$\bar{1}\;\bar{1}$  ) Bragg-reflection in diamond, the scattering angle is 2*θ* = 35.04^◦^ for 10.1 keV X-rays. In this setting, the effective pixel size in the illuminated plane is 215.8 nm × 375.9 nm along *y_ℓ_* and *z_ℓ_*, respectively. A region-of-interest was set corresponding to a field of view of the microscope within the illuminated layer of 221 µm × 385 µm. The FWHM thickness (along *x_ℓ_*; see Fig. 1) of the observation plane formed by the incident X-rays was Δ*x* ≈ 3.9 µm. The spatial resolution in the two orthogonal directions is much better, essentially given by the detector pixel size. For details of the spatial and angular resolution, see *SI Appendix*, section 5. Weak-beam contrast was used with an angular offset of +0.0764 mrad in *φ* with respect to the orientation where the strain-free parts of the diamond crystal are in the Bragg condition.

Images were acquired with the X-ray probe beam having time delays of Δ*t* with respect to the arrival time of the optical pump. The signal-to-noise ratio in individual images is ∼8.5. To improve signal-to-noise, we collected 240 frames at each time delay. By default, the images in this work (and subsequent datasets) represent the average of the 240 frames per time step, corrected for background. An example of such a set of individual frames is provided as Movie S1. No beam damage was observed while exposing a fixed volume for 3 h with the LCLS repetition rate of 120 Hz.

In Fig. 2 and *SI Appendix,* Fig. S2, there is a jump in the position of strain-wave B between 11 and 12 ns. This was due to an anomalously large time step and not a real effect.

## Supplementary Material

Appendix 01 (PDF)

Movie S1. Gallery of 120 randomly selected raw images for a time delay of 77.97 ns. This illustrates the intensity fluctuations between individual X-ray pulses.

Movie S2. DFXM movie of the structural changes in observation plane during the first 100 ns after the ultrafast heating of the Au foil.

Movie S3. DFXM movie of the structural changes in observation plane during the first 1800 ns after the ultrafast heating of the Au foil. Shown are snapshots acquired at times 5.5 ns + nΔ*t_p_*, where *n* is an integer and Δ*t_p_* = 72.47 ns is the period corresponding to the fast strain wave travelling from the Au coated surface to the free surface and back.

## Data Availability

All study data are included in the article and/or supporting information. Raw data and code have been deposited in Zenodo (https://zenodo.org/record/8303112) (37).
