# Supplementary material for: Real-time imaging of acoustic waves in bulk materials with X-ray microscopy
Source: Proc Natl Acad Sci U S A. 2023 Sep 19;120(39):e2307049120. doi: 10.1073/pnas.2307049120 (PMC10523471; doi:10.1073/pnas.2307049120)
Supplement: Supplementary file 1 — Appendix 01 (PDF) [file pnas.2307049120.sapp.pdf]

Supplementary materials

Supplementary Text including Figs. **S1** to **S11**. Movies
M1 to M3.

1. Real-time X-ray microscopy movies of the acoustic waves

Movie M1: Gallery of 120 randomly selected raw images for a time delay of 77.97 ns. This illustrates the intensity fluctuations between individual X-ray pulses.

Movie M2: DFXM movie of the structural changes in observation plane during the first 100 ns after the ultrafast heating of the Au foil.

Movie M3: DFXM movie of the structural changes in observation plane during the first 1800 ns after the ultrafast heating of the Au foil. Shown are snapshots acquired at times 5.5 ns + $n\Delta t_p$, where n is an integer and Δt_p = 72.47 ns is the period corresponding to the fast strain wave travelling from the Au coated surface to the free surface and back.

2. Geometry of the experiment

The geometry of the experiment is sketched in Fig.1. The X-ray beam illuminates a 2D-sheet (the observation plane) of the diamond crystal, while the strain waves have an anisotropic three dimensional structure. In Figure **S1** we provide a schematic of the hypothesized 3D profiles of the two strain waves, for a given time delay, assuming the waves travel in a homogeneous media. The figure illustrates that the waves are near planar within the central part of the field of view - consistent with the DFXM images.

Figure **S2** displays the evolution of the intensity profiles (averaged over y_ℓ) of the two strain waves in Fig. 2 during the initial 30 ns. The intensity profiles are seen to be relatively constant over the initial 150 µm, corresponding to the diameter of the laser spot on the surface. After 150 µm, the strain wave intensity decreases as z_ℓ^{-2}, consistent with the energy being distributed over a spherical surface.

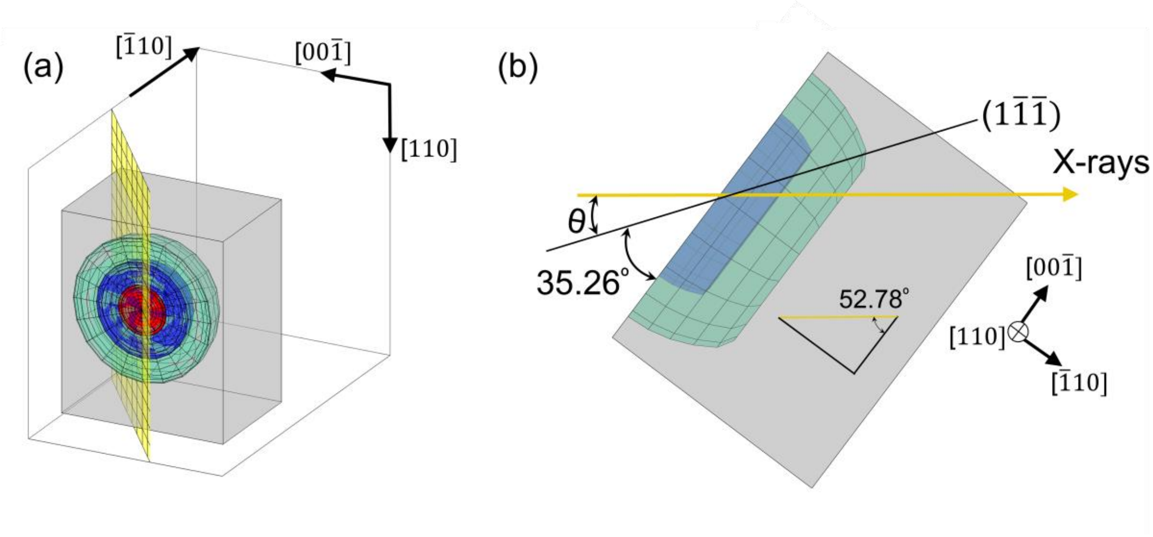

Figure **S1**: 3D Schematic of the geometry of the experiment. The coordinate systems are the same as in Fig. 1. (a) The transparent blue and aquamarine surfaces represent the transverse and longitudinal acoustic strain waves, respectively. The red area represents the 150 µm optical laser spot on the Au-coated surface. Within a region given by the optical laser spot size the strain waves travel as planar waves. (b) Top-down view on the the diamond crystal, which is oriented such that Bragg-scattering occurs from the $(1\bar{1}\bar{1})$-planes. Hence, the angle between the observation plane (the layer illuminated by the incident X-ray beam; yellow transparent surface in this figure) and the $(1\bar{1}\bar{1})$-planes is θ = 17.52°. The angle between $(1\bar{1}\bar{1})$-planes and the $(\bar{1}10)$-planes is 35.26°. Thus, the relation between the observed distance in the observation plane z_ℓ and the actual distance z_{sw} the waves travel along $[\bar{1}10]$ is given by $z_{\mathrm{sw}} = z_\ell \cdot \sin(52.78^\circ)$.

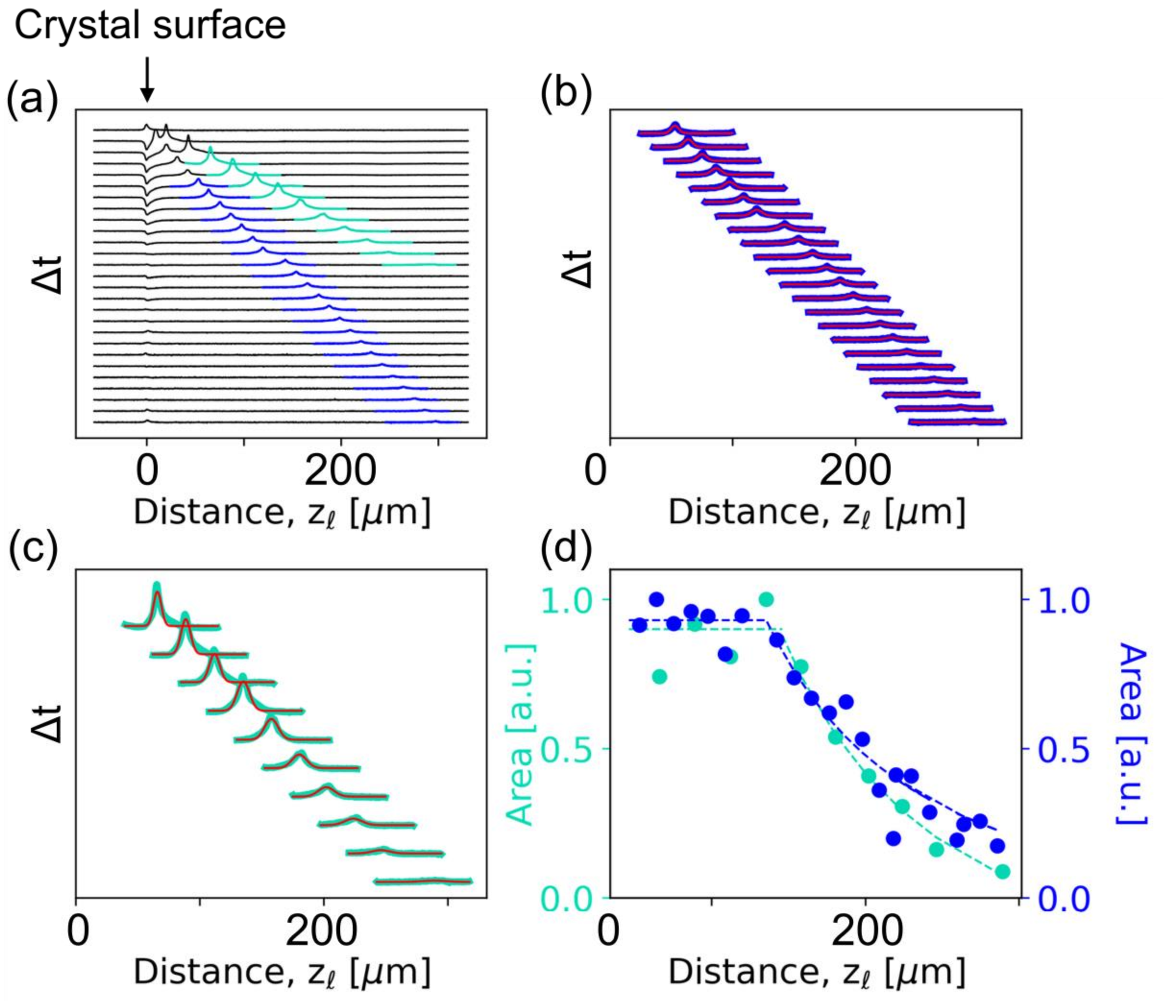

Figure **S2**: (a) Intensity curves (averaged over y_ℓ) as function of distance z_ℓ (same data as in Fig. 2). (b,c) Gaussian fits (red curves) to the experimental data for strain waves B and A, respectively. (d) The areas of the Gaussian fits to peak B and peak A are plotted as function of distance (dots). In both cases data are normalised to 1. The dashed lines are best fits to models, where areas are constant in the initial 150 µm, and then decreases as z_ℓ^{-2} for larger z_ℓ.

3. Transfer of energy from the longitudinal to the transverse wave

We observe that for all periods n, the strain wave A is reflected upon the Au-deposited surface, and generating a new slow strain wave B_n in the process (the same process may occur on the opposite surface, but this was outside of the field of view, and thus not directly observed). As the intensity profiles of the B_n waves are identical as function of time delay since their creation, we infer that they are all transverse waves of the same type as B_1.

We make the hypothesis that the creation of transverse waves in each period is associated with a transfer of a fixed fraction of energy from the longitudinal wave to the new transverse waves. As illustrated in Fig. **S3** a combined fit to the decay of the longitudinal wave and the decaying relative intensities of the new transversal waves corroborates this hypothesis. The fitted values are provided in Table **S1**. The fitted decay rate b = 0.116 corresponds to a constant transfer of energy from the longitudinal to the transverse waves and a "reflectivity" for each period of $R = e^{-b} = 0.89$.

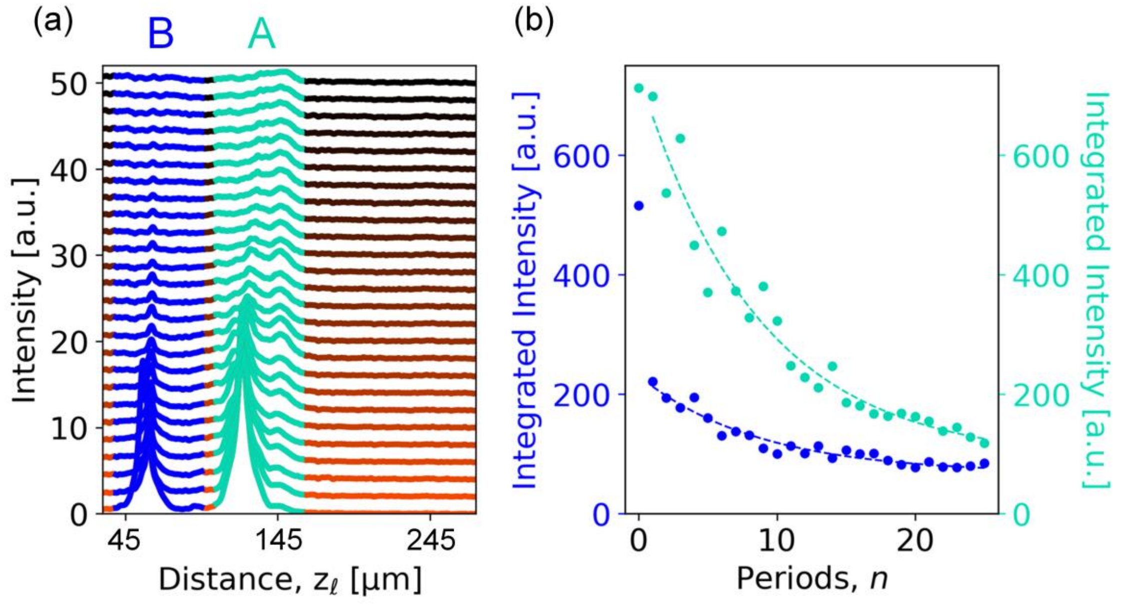

Figure **S3**: (a) Intensity profiles for 26 equi-distant time delays of Δt = 5.5 ns $+n\Delta t_p$, where n is an integer and $\Delta t_p = 72.47$ ns is the time period it takes for the longitudinal strain wave A to travel from the Au-coated surface to the free surface and back. The curves are offset by $2n$*a.u. for ease of visualisation. The curves are integrated within the regions $z_\ell \in [39\ 95]$ μm and $z_\ell \in [105\ 161]$ μm for peak B and A, respectively (indicated by color). (b) The resulting integrated intensities as a function of number of periods, n (dots). The two dashed lines represent a simultaneous fit to functions $I_i = a_i e^{-bn} + c_i$, i = A, B with c_i being background terms. The quality of this fit corroborates a hypothesis that both peaks exhibit exponential decays with the same constant $b = 0.116$.

Table **S1**: The optimised parameters a_i, b and c_i, based on the fit illustrated in Fig. **S3**.

i	A	B
a_i	646.2	163.2

b	0.116	0.116
c_i	89.8	67.5

4. Mapping strain components

DFXM is sensitive to the individual components of the displacement gradient tensor field $\mathbf{F}(\vec{r},t)$ (Ref. 1), which can be expressed in terms of an orientation field $\mathbf{\Omega}(\vec{r},t)$, characterizing grains and domains, and an elastic strain tensor field, $\epsilon(\vec{r},t)$, related to the local stresses by Hooke's law.

With the set-up illustrated in Fig. 1, where we diffract from the $\vec{Q}$ = $(1\bar{1}\bar{1})$ lattice planes, contrast can be provided in three ways (see Fig. 1):

- *Rotation around* y_ℓ by angle ϕ. Known as a rocking scan, this probes a shear strain: the displacement of $\vec{Q}$, along the $[\bar{1}1\bar{2}]$ direction. (For a longitudinal wave along $[\bar{1}10]$ the component of the strain ϵ_L that displaces $\vec{Q}$ along direction $[\bar{1}1\bar{2}]$ is ϵ_L cos(54.74°), ° is the angle between $[\bar{1}10]$ and $[1\bar{1}2]$. This is the contrast mode used in Figs. 1-3, **S2**-**S3**, and **S7**-**S10**).
- *Rotation around* z_ℓ by angle χ. Known as a rolling scan, this probes another shear strain: the displacement of $\vec{Q}$ along the $[110]$ direction.
- *Variation of axial strain*. A combined $2\theta - \phi$ scan probes the axial strain (the elongation) in direction $\vec{Q}$.

A longitudinal wave travelling in direction $[\bar{1}10]$ will exhibit strain in the same direction. If the instrumental blurring is negligible this is visible in both ϕ and axial strain scans, while χ-scans are not sensitive to this strain component. The same is true for a transverse wave travelling in direction $[\bar{1}10]$ with a strain in direction $[00\bar{1}]$. On the other hand a transverse wave travelling in direction $[\bar{1}10]$ with a strain in direction $[110]$ provides only contrast when χ is offset from 0. With a more quantitative description[1] it appears that DFXM can identify both the direction of propagation and the direction of the displacement of the acoustic waves.

Due to mechanical constrains in the *ad hoc* setup, χ and 2θ could not be varied. What can be deduced is that the observation by ϕ-contrast of the two strain waves is consistent with a longitudinal wave and a slow transverse wave (with a displacement in direction [00$\bar{1}$]) as suggested by the speed of sound values. From this follows that a third fast transverse wave (with displacement in direction [110]) might be created, but is invisible with the configuration used.

5. Comparing experimental and simulated strain-wave profiles

In this section we will compare experimental DFXM images to geometrical-optics-based forward modelling projections. The forward model takes a thermomechanical model of the strainwave as its input. The thermomechanical model for ultrafast dynamics applied, udkm1Dsim[2], is one-dimensional and represents a longitudinal wave. The strain profile arising for a 300 nm Au film on 15 nm Ti on diamond, excited by a 100 μJ optical laser pulse with a 150 μm FWHM diameter spotsize on the sample surface, is presented in Fig. **S4**. Once the strain wave is formed its profile does not change with time in this model (no dispersion).

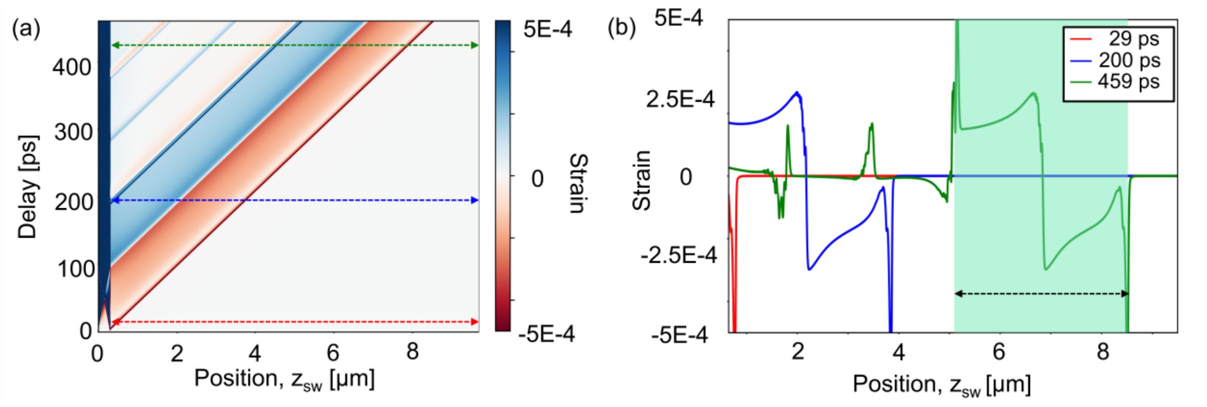

Figure **S4**: Strain-wave profile in a diamond single crystal as a function of time delay from laser pulse heating, as computed using a 1D thermomechanical model. (a) 2D map of strain versus depth and time delay. (b) 1D plots of the strain profile in diamond at different time delays (indicated with dashed lines in (a)). The spatial extent of the part of the strain wave that is visible in DFXM at 459 ps (indicated in transparent green) is about 3.5 µm.

The DFXM forward projection model used is a simple adaptation of the geometrical optics code presented in Ref. 1. This uses a synchrotron convention for the laboratory coordinate system[1,3]. Its relation to the XFEL laboratory coordinate systems used in this work can be seen by comparing Figs. 1 (a,b) and **S5** (a,e), and is given by

$$\mathbf{r}_{\ell,\text{XFEL}} = \begin{bmatrix} 0 & 0 & 1 \\ 0 & -1 & 0 \\ 1 & 0 & 0 \end{bmatrix} \mathbf{r}_{\ell,\text{sync.}} \qquad (1)$$

In the geometrical optics formalism, several coordinate systems are used[1,3]. The sample and the grain coordinate systems are defined in Fig. **S5**. The sample coordinate system is rotated an angle θ relative to the laboratory coordinate system. The grain coordinate system is defined according to the crystallographic directions in the diamond crystal. An additional

strain-wave coordinate system is defined to have z_{sw} along the propagation direction of the strain wave, $[\bar{1}10]_g$, and x_{sw} along $[001]_g$.

Let us assume a *longitudinal* wave propagating along $[\bar{1}10]_g$. Given the 1D thermomechanical model the displacement gradient field, $\mathbf{F}(\vec{r},t)$ has only one non-trivial component:

$$\mathbf{F}_{sw} = \begin{bmatrix} 1 & 0 & 0 \\ 0 & 1 & 0 \\ 0 & 0 & 1 + f(zsw) \end{bmatrix}, \tag{2}$$

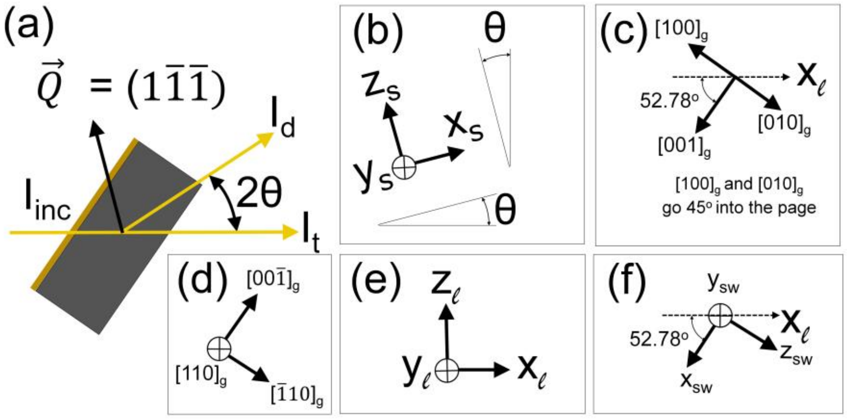

Figure **S5**: Coordinate systems used, with reference to the conventions used in Ref. 1. (a) Experimental geometry (horizontal plane) with $\vec{Q}$ the diffraction vector and I_{inc}, I_d and I_t being the incoming, diffracted and transmitted beams, respectively. (e) The laboratory coordinate in the synchrotron convention used in Ref. 1. (In the rest of the paper, outside this section, with direct reference to the geometrical optics model, we use the XFEL convention.) (b) The associated sample coordinate system rotated θ around the y_ℓ-axis relative to the laboratory coordinate system. (c) The grain coordinate system projected down on the plane of the figure (the x- and y-axes go 45° into the page). (d) The grain coordinate system, with directions parallel to the diamond crystal's facets. (f) An additional strain-wave coordinate system, with x_{sw} along $[001]_g$ and z_{sw} along $[\bar{1}10]_g$.

where $f(z_{sw})$ is the strain-profile in Fig. **S4**. $\mathbf{F}_{sw}$ is related to $\mathbf{F}_g$ by equation (75) in Ref. 1:

$$\mathbf{F}_g = \mathbf{U}_{sw}\mathbf{F}_{sw}\mathbf{U}^T_{sw}, \tag{3}$$

with the coordinate transform

$$\mathbf{r}_g = \mathbf{U}_{sw}\mathbf{r}_{sw}. \tag{4}$$

Figure **S5** shows the relation between the grain coordinate system and the strain wave coordinate system. x_{sw} lies along $[001]_g$, y_{sw} lies along $[110]_g$ and z_{sw} lies along $[\bar{1}10]_g$. The columns of $\mathbf{U}_{sw}$ are the basis vectors in the strain-wave coordinate system expanded in the basis of the grain coordinate system. Hence,

$$\mathbf{U}_{sw} = \begin{bmatrix} 0 & \frac{1}{\sqrt{2}} & -\frac{1}{\sqrt{2}} \\ 0 & \frac{1}{\sqrt{2}} & \frac{1}{\sqrt{2}} \\ 0 & 0 & 0 \end{bmatrix}. \quad (5)$$

The orientation of the diamond crystal $\mathbf{U}$ enters into the formalism by the definition of the "grain" system Ref. 1:

$$\mathbf{r}_s = \mathbf{U}\mathbf{r}_g. \quad (6)$$

In Fig. **S5**, it can be seen that x_s is parallel to $[110]_g \times [1\bar{1}\bar{1}]_g = [\bar{1}1\bar{2}]_g$, y_s is parallel to $[110]_g$ and z_s is parallel to $[1\bar{1}\bar{1}]_g$. The columns of $\mathbf{U}$ are the basis vectors in the grain coordinate system expanded in the basis of the sample coordinate system. Hence,

$$\mathbf{U}_{sw} = \begin{bmatrix} -\frac{1}{\sqrt{6}} & \frac{1}{\sqrt{6}} & -\frac{2}{\sqrt{6}} \\ \frac{1}{\sqrt{2}} & \frac{1}{\sqrt{2}} & 0 \\ \frac{1}{\sqrt{3}} & -\frac{1}{\sqrt{3}} & -\frac{1}{\sqrt{3}} \end{bmatrix}. \quad (7)$$

Both $\mathbf{U}$ and $\mathbf{U}_{sw}$ satisfy $\mathbf{A}^T = \mathbf{A}^{-1}$ and $\det(\mathbf{A}) = 1$.

Angular and Spatial resolution

The geometrical optics formalism introduced above simulates the 6D reciprocal space-direct space resolution function[1]. This was used to optimize the set up prior to actual beamtime[4]. For reference, we here provide results for the parameters actually used during experiment.

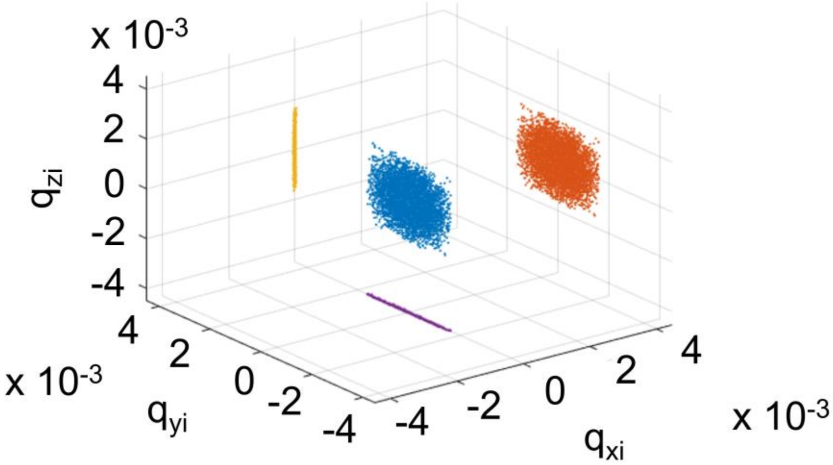

Figure **S6**: Reciprocal space resolution function for the DFXM set up at LCLS. The simulation presented (for visualisation purposes) involved 10000 simulated rays. Dark blue cloud at center: 3D scatter plot for the resolution function expressed in the imaging coordinate system. The purple, orange and yellow symbols correspond to 2D projections onto the q_{xi}-q_{yi} plane, q_{yi}-q_{zi} plane and the q_{xi}-q_{zi} plane, respectively. (x_i, y_i, z_i) denotes the imaging coordinate system (see text, and Refs. 1,3).

Reciprocal space resolution function. A Monte Carlo ray simulation of the reciprocal space resolution function is visualised in Fig. **S6**. (The imaging coordinate system is used, which corresponds to the laboratory coordinate system rotated by 2θ around y_ℓ (Refs. 1, 3)). 10000 rays were used in Fig. **S6** to visualize the anisotropy. Comparison of the projection shown in orange with those in yellow and purple shows a large anisotropy in the resolution function. To first order, the resolution function is a disc, with a “thin dimension” parallel to the optical axis of the objective. The dimensions of the two wide axes are defined by the acceptance functions set by the numerical aperture of the objective, NA, producing a nearly planar distribution. The NA is larger than the maximum strain in the acoustic waves. In the actual simulations, 100 million rays were used for accuracy, filling 500 points in a range of 2.5E-3 along the three axes in reciprocal space. The energy band width (FWHM) was $\Delta E/E \sim 10^{-4}$, the divergence (FWHM) $\Delta\zeta$ = 30 µrad in both horizontal and vertical directions. In the objective CRL, the lenslets had a radius of curvature of R = 50 µm, and a center-to-center distance between successive lenslets T = 1 mm. The sample-to-objective-entry plane distance was $d_1 = 0.23$ m. The NA was 3.598E-04 (root-mean-square).

The majority of the crystal is strain free and will therefore give rise to diffraction at the Origo with $\vec{Q} = \vec{Q}_0$. The sound wave may be visible in this "strong beam" but dynamical diffraction makes it difficult to quantify such images. For this reason, in this experiment "weak beam contrast" is applied, implemented by a rotation in ϕ. The range of the strong beam condition is given by the width (FWHM) of the plate - this is according to the simulations $\Delta\phi < 10^{-4}$, as shown in Fig. **S10** (c).

Direct space resolution function. The spatial resolution function is anisotropic, dominated by the beam width. To illustrate the effect, Fig. **S7** shows the intensity-profile across a strainwave (integrated over y_ℓ) resulting from having a Heaviside step-function along z_{sw} in the geometrical optics simulation. The offset in ϕ was set to +0.0764 mrad (the offset in ϕ is defined relative to the center of mass of the rocking curve in the bulk; see Fig. **S9** (c) below).

$$\mathbf{F}_{sw} = \begin{bmatrix} 1 & 0 & 0 \\ 0 & 1 & 0 \\ 0 & 0 & 1 - A \cdot H(z\mathrm{sw}) \end{bmatrix}, \quad (8)$$

where A is the amplitude of the perturbation, here chosen to be 2E-4 to reflect Fig. **S4**. In Fig. **S7**, the negatively strained part of the crystal (for $z_{sw} > 0$) comes into the Bragg condition for $\phi > 0$, an observation that will be important in the analysis of rocking scans below.

The derivative of the step-function represent the instrumental blurring associated with a strain wave propagating along z_{sw}. In the two orthogonal directions the spatial resolution is to a first approximation given by the effective pixel size within the sample, as the angular contributions arising from the divergence of the incident beam and the energy bandwidth are small.

Comparison of intensity profiles at fixed offset in ϕ

Figure **S8** (a) shows a simulated DFXM image for an angular offset in ϕ of +0.0764 mrad (the offset in ϕ is defined relative to the center of mass of the rocking curve in the bulk; see Fig. **S9** (c) below). A comparison of this and a line-out representing an integration in the vertical direction of the image with the experimental data in Fig. **S8** (b) shows a satisfactory correspondence. The maximum intensity in the raw individual images is about 200 counts/pixel, cf. Movie 1. The maximum intensity in Fig. **S8** (a) is ~210 counts/pixel (using the noise model in Ref. 4, and assuming a third of the 1.6mJ/10.1keV = 9.9e11 photons make it past the monochromator). The strain wave in Fig. **S8** (b) was taken from a position within the first 150 µm to avoid probing the curved part of the wave-front in Fig. **S1**.

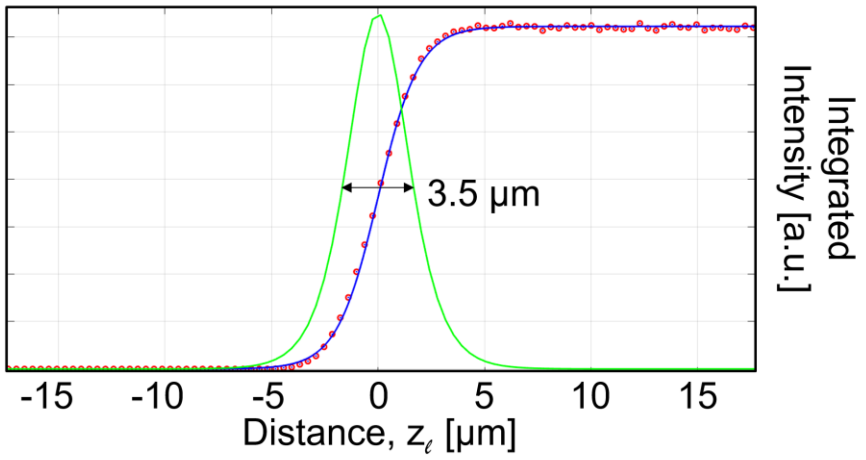

Figure **S7**: Simulated instrumental blur along z_ℓ for ϕ = +0.0764 mrad. Modelling the deformation gradient tensor field as a Heaviside function in z_{sw} the geometrical optics forward simulator produces an intensity profile shown with red dots. A sigmoid function was fitted to this intensity profile (blue line). The FWHM of the derivative (green line) is 3.5 µm.

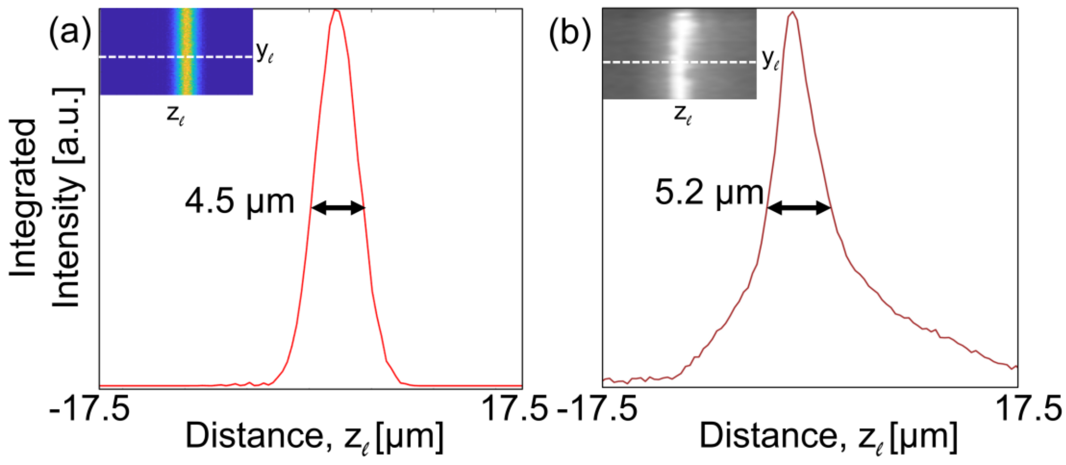

Figure **S8**: Comparison of profiles of the fast strain wave for an offset in ϕ from the strong beam condition by +0.0764 mrad. (a) As simulated by a combined thermomechanical and X-ray geometrical optics forward simulation[4]. The simulation relates to a planar longitudinal acoustic wave travelling in direction $[\bar{1}10]_g$. The integrated line profile of the peak is shown. The inset shows the full DFXM image with the z_ℓ-axis marked by a dashed line. Here yellow signifies maximum intensity and dark blue minimum intensity. (b) Corresponding experimental DFXM data.

Comparison of rocking scans

By varying ϕ we probe one shear strain component. The strain sensitivity is essentially given by the "thin" direction of the reciprocal space resolution function introduced in Fig. **S6**. Experimentally a ϕ rocking-scan was performed at a delay of Δt = 5.5 ns, in steps of $\Delta\phi$ = $1.309 \cdot 10^{-5}$ rad. The resulting movie is summarised in Fig. **S9**, displaying the intensity (averaged over y_ℓ) as function of z_ℓ horizontally and ϕ vertically. The rocking curve at distances far from the strain wave is seen as representing the combined Darwin width and instrumental resolution function, and we set ϕ = 0 at center of mass position (Fig. **S9** (c)). In the vicinity of the fast strain wave, the distribution is asymmetric with two lobes, see Fig. **S9** (a). The center of mass of these are separated by $\sim$1 µm along z_ℓ and by and $\sim$0.05 mrad in ϕ.

Notably the minimum intensity between these two is shifted by $1.963 \cdot 10^{-5}$ rad in relation to ϕ = 0, as defined by Fig. **S9** (c). We speculate this is caused by a slight rotation of the lattice. We also note that the maximum intensity in sub-figure (a) is much higher than in (c). We attribute this to dynamical diffraction effects, to be explored in future studies.

To further understand the data in Fig. **S9**, we forward simulated DFXM images of the strain waves at different ϕ values (100 values in the range ± 0.2 mrad; $\Delta\phi$ = $4.04 \cdot 10^{-6}$ rad) using the 1D thermomechanical model to generate the strain waves in Fig. **S4** and the geometrical optics simulation tools already described. From these simulations, we generated 2D plots similar to those of Fig. **S9**. The result can be seen in Fig. **S10**.

Figure **S10** (a) is scaled to a maximum of 415 counts per pixel. Two lobes can be seen, with centers of mass that are displaced in a qualitatively similar fashion to the lobes in the experimental data. However, quantitatively the separation-distances between the centers of mass of $\sim$0.1 mrad in ϕ, and $\sim$2 µm along z_ℓ, are both significantly larger than the separation between the centers of mass in the experimental data in Fig. **S9** (a). We speculate that this reflects effects of dynamical diffraction, uncertainties in the model parameters and/or inadequacies related to using a 1D thermomechanical strain-wave model for a 3D process (see Fig. **S1**).

Figure **S10** (b) is scaled to a maximum of 1000 counts per pixel, corresponding to the strongbeam condition. In the simulation, the position of ϕ = 0 (defined by taking the center of mass of the rocking-curve in Fig. **S10** (c)) is symmetric between the two strain-wave maxima.

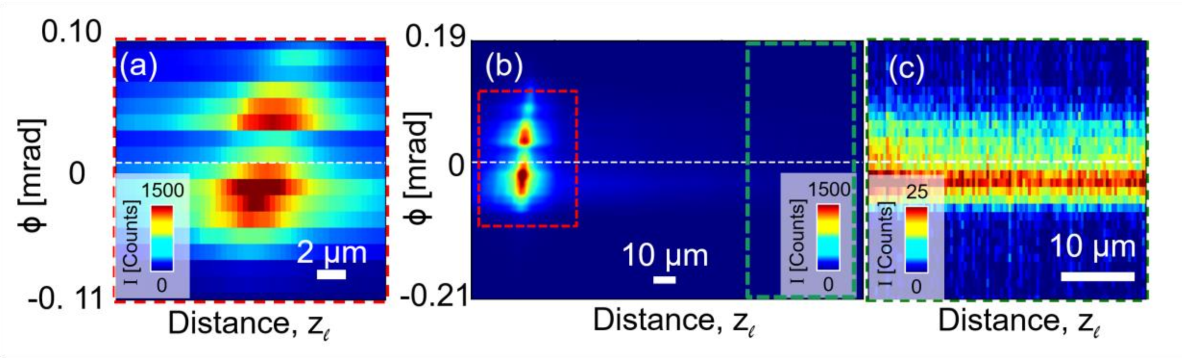

Figure **S9**: Experimental ϕ -scan across a longitudinal strain-wave. (a) Zoom-in on the position of the strain-wave, showing two horizontally displaced strain-wave positions for different values of ϕ. (b) Zoomed out overview. (c) Zoom-in on the bulk region marked with dashed green lines in (b), showing the bulk rocking-curve. The dashed white line describes the center of mass of the bulk rocking-curve, which we take as ϕ = 0.

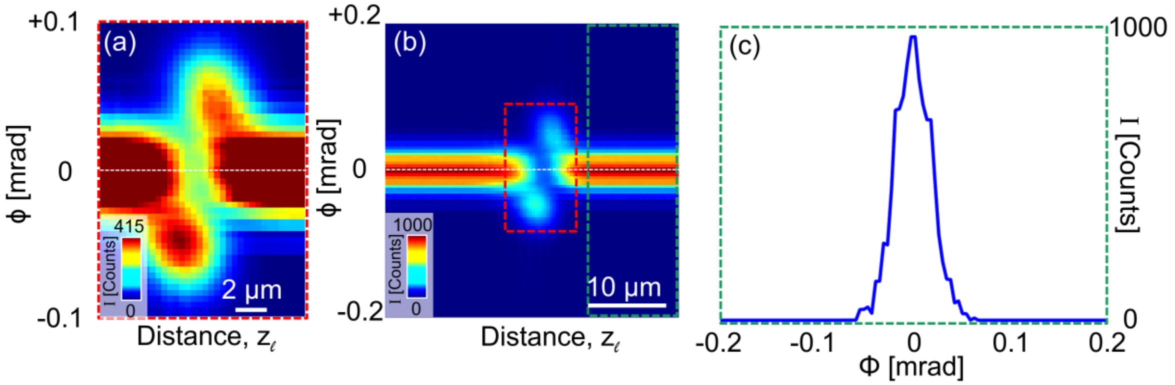

Figure **S10**: Geometrical optics simulated plot of ϕ-scan across a longitudinal strain-wave. (a) Scaled to show the weak-beam. (b) Scaled to show the strong-beam. (c) Plot of rocking curve in bulk averaged over z_ℓ in the region marked with green dashed lines in (b).

Smearing due to beam-thickness

The aim of this section is to provide an intuitive understanding of the effect of the beam thickness on the strain-wave measurements. The FWHM thickness along x_ℓ is 3.9 µm. Projected onto the $[\bar{1}10]_g$ direction this corresponds to 3.9 · cos(52.78°) µm ≈ 2.4 µm.

Assume a box shaped smearing function with this FWHM. In Fig. **S11** (a) we plot a histogram of the resulting strain values as function of position for a simple strain wave model (red curve),

$$f(z_{\mathrm{sw}}) = -\frac{A}{s} \cdot (z_{\mathrm{sw}} - z_0) \cdot e^{\frac{(z_{\mathrm{sw}}-z_0)^2}{2s^2}}, \tag{9}$$

where A is the amplitude of the strain wave, s a parameter describing its spatial width, and z_0 the central position. The overlaid colormap is a plot of the histograms. Next, the same histogram representation is made based on the one-dimensional thermomechanical model in Fig. **S4**, resulting in Fig. **S11** (b).

Figure **S11** shows that when the strain-profile is convoluted with the beam-thickness, a given part of the strain-wave profile can be detected in multiple positions of the box-beam, and when the strain-profile becomes flat, e.g. at the extreme positions, similar strain-values add up, leading to a maximum in the histograms. In the experimental data, this would manifest as maxima in the rocking-curve data, such as those seen in Figs. **S9** (a) and **S10** (a) (when the comparison is made it must be remembered that negative (positive) strain along $[\bar{1}10]_g$ leads to the Bragg-condition shifting to $\phi > 0$ ($\phi < 0$), as noted in the discussion of Fig. **S7**).

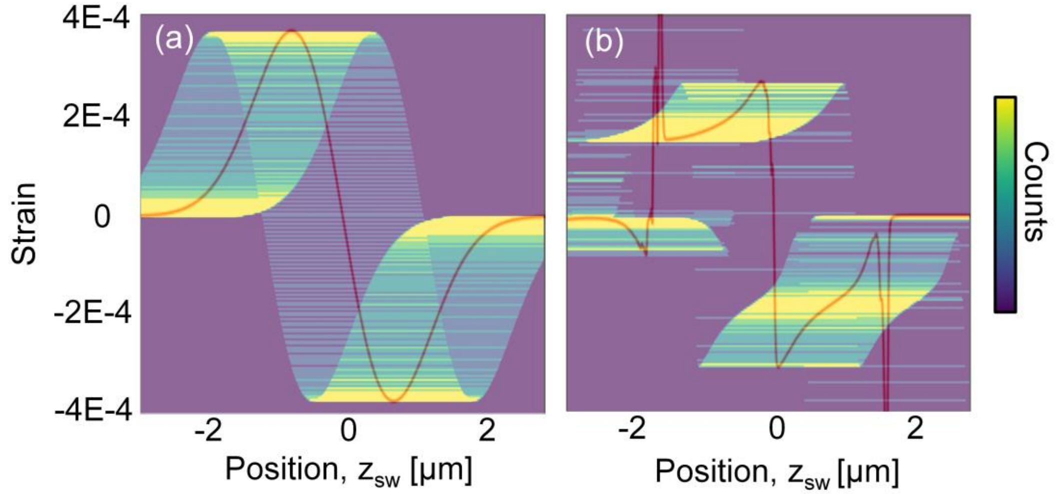

Figure **S11**: Effect of beam width on strain profile using as models for the longitudinal strain wave (red lines) (a) an idealized model, see text, and (b) a 1D thermomechanical simulation. In both cases the profiles are smeared out by a box-function of width 2.4 µm. The overlaid colormaps are the resulting intensity profiles.

6. Calculating the velocities of the acoustic waves

The sound velocity for strain wave A can be determined with high accuracy by the time Δt it takes to perform 25 periods and the sample thickness (660 µm).

$$v = \frac{(660\ \mu\mathrm{m} \cdot 2) \cdot 25}{(1817.25 - 5.5)\ \mathrm{ns}} = 18.21\ \mathrm{km/s}$$

From the slopes in Fig. 2, it is established that the velocity of strain wave B is 48.6 % of the velocity of strain wave A, that is to say 8.86 km/s.

References

1. Poulsen, H. F., Dresselhaus-Marais, L. E., Carlsen, M. A., Detlefs, C. & Winther, G. Geometrical-optics formalism to model contrast in dark-field X-ray microscopy. *J. Appl. Crystallogr.* **54**, 1555 (2021).

2. Schick, D. udkm1Dsim – a Python toolbox for simulating 1D ultrafast dynamics in condensed matter. *Comput. Phys. Commun.* **266**, 108031 (2021).

3. Poulsen, H. F., Jakobsen, A. C., Simons, H., Ahl, S. R., Cook, P. K. & Detlefs, C. X-ray diffraction microscopy based on refractive optics. *J. Appl. Cryst*. **50**, 1441 (2017).

4. Holstad, T. S. *et al.* X-ray free-electron laser based dark-field X-ray microscopy: a simulation-based study. *J. Appl. Crystallogr.* **55**, 112 (2022).
